# Three-Dimensional Imaging and Modeling of Anatomic Structures, Sectional and Radiological Anatomy, and Staining Techniques

**DOI:** 10.1155/2012/970585

**Published:** 2012-02-29

**Authors:** Tuncay Peker, Nadir Gülekon, Ilkan Tatar, Levent Sarıkcıoğlu, David Kachlik

**Affiliations:** ^1^Department of Anatomy, Gazi University, Faculty of Medicine, 06500 Ankara, Turkey; ^2^Department of Anatomy, Hacettepe University, Faculty of Medicine, 06100 Ankara, Turkey; ^3^Department of Anatomy, Akdeniz University, Faculty of Medicine, 07058 Antalya, Turkey; ^4^Department of Anatomy, Third Faculty of Medicine, Charles University, 100 00 Prague 10, Czech Republic

The first three-dimensional (3D) reconstruction was performed by Born (1883) and His (1885) using serial sections. Since then, a variety of reconstruction techniques have been developed, and the construction of physical models has become important in anatomy. The first computer-aided 3D reconstruction was achieved by Glaser and Van der Loos in 1965. With the improvements in both computer hardware and software tools, computerized modelling of anatomical structures has become very useful for visualizing complex 3D forms. Three-dimensional visualization of various microanatomic structures using special preparation and staining methods is important. Devices used for this purpose such as MicroCT, microMRI, SEM, and Confocal microscopy and so forth. are development parallel to the technological advances and give excellent 3D images, and it allows a better understanding of gross and microanatomic structures. On the other hand, first studies on cross-sectional anatomy date back to the 16th century. The widespread use of cross-sectional imaging techniques such as CT and MRI has gained an increasing importance in sectional anatomy. The most known cadaveric cross-sectional study, the visible human project, is an outgrowth of the National Library Medicine.

In this special issue, we reviewed and edited seven articles on three-dimensional imaging and modeling of anatomic structures, sectional and radiological anatomy, and staining techniques. 

K. K. Schmid (University of Nebraska, USA) Marx and Samal (University of Nebraska-Lincoln, USA) discussed three-dimensional regression, a technique that can be used for mapping images and shapes that are represented by sets of three-dimensional landmark coordinates, for comparing and mapping 3D anatomical structures in their research paper.

Y. Hoshino and coworkers (University of Pittsburgh, USA) reviewed previous research about image analysis of the anterior cruciate ligament (ACL) anatomy and its application to ACL reconstruction surgery. They concluded that three-dimensional image analysis of the ACL anatomy and its application to the navigation system is becoming more prevalent and reliable for advancing the anatomic studies related to the native ACL and the ACL reconstruction procedure.

A. Arencibia et al. (Veterinary Faculty, University of Las Palmas de Gran Canaria) investigated imaging features of the temporomandibular joint (TMJ) using computed tomography and magnetic resonance imaging in two normal camels. They stated in conclusion that CT is an excellent method for the detailed assessment of the bony structures, and MRI is a valid imaging modality for the evaluation of the soft tissues. Processing of CT and MR images allowes to appreciate different bone structures and soft tissues of the TMJ, assisting in the interpretation of the images.

J. A. N. Buytaert and colleagues (University of Antwerp and Ghent University, Belgium) discussed the first implementation of (laser) light-sheet-based fluorescence microscopy (LSFM) to image biomedical tissue in three-dimensional orthogonal plane fluorescence optical sectioning microscopy (OPFOS). They have shown with several applications that the OPFOS (and derived) methods are a valuable addition for sectional imaging and three-dimensional modeling of anatomic structures.

The review paper entitled “*Ultrahigh voltage electron microscopy (UHVEM) links neuroanatomy and neuroscience/neuroendocrinology*” by H. Sakamoto (Okayama University, Japan) and M. Kawata (Kyoto Prefectural University of Medicine, Japan) stressed that UHVEM and light microscopy are useful and powerful tools for studying molecular and/or chemical neuroanatomy at the ultrastructural level.

D. R. Marker et al. (The Johns Hopkins Hospital and University School of Medicine, Weill Cornell Medical College, USA) discussed strategic improvements for gross anatomy web-based teaching in their research paper. To better meet the expectations of gross anatomy students, electronic radiology teaching files for first-year coursework were organized into a web site. The web site was custom designed to provide material that directly correlated to the gross anatomy dissection and lectures. Quick links provided sets of images grouped by anatomic location. Their findings suggest that a well-organized web portal can provide a user-friendly, valuable educational resource for medical students who are studying gross anatomy.

Finally, Herber and coworkers (University of California, USA) in their study entitled “*Imaging an adapted dentoalveolar complex*” used micro-X-ray computed tomography combined with 3D modeling using image processing, scanning electron microscopy, fluorochrome labeling, conventional histology (H&E, TRAP), and immunohistochemistry (RANKL, OPN) to elucidate the dynamic nature of bone, the periodontal ligamentspace, and cementum in the rat periodontium. They concluded that studies focusing on a functional rat dentoalveolar complex should acknowledge the baseline phenomena of bone-PDL-cementum adaptation, especially in regards to the physiological distal drift, before additional experimental variables are imposed.

We hope that this special issue will contribute as a stimulus for further research into three-dimensional imaging and modeling of anatomic structures, sectional and radiological anatomy, and staining techniques. We are very grateful to the contributing authors for their scientific contributions to this special issue. We also thank the reviewers who spend their valuable time and thoughts on each paper. We appreciate the Hindawi Publishing Corporation for their help in the guidance of the procedure. 

*Tuncay Peker* *Nadir Gülekon* *Ilkan Tatar* *Levent Sarıkcıoğlu* *David Kachlik*

